# Reciprocal signaling between adipose tissue depots and the central nervous system

**DOI:** 10.3389/fcell.2022.979251

**Published:** 2022-09-16

**Authors:** Stephanie C. Puente-Ruiz, Alexander Jais

**Affiliations:** Helmholtz Institute for Metabolic, Obesity and Vascular Research (HI-MAG) of the Helmholtz Zentrum München at the University of Leipzig and University Hospital Leipzig, Leipzig, Germany

**Keywords:** central nervous system, hypothalamus, adipose tissue, sympathetic regulation, lipolysis, adipogenesis, adipose tissue macrophage, resident immune cells

## Abstract

In humans, various dietary and social factors led to the development of increased brain sizes alongside large adipose tissue stores. Complex reciprocal signaling mechanisms allow for a fine-tuned interaction between the two organs to regulate energy homeostasis of the organism. As an endocrine organ, adipose tissue secretes various hormones, cytokines, and metabolites that signal energy availability to the central nervous system (CNS). Vice versa, the CNS is a critical regulator of adipose tissue function through neural networks that integrate information from the periphery and regulate sympathetic nerve outflow. This review discusses the various reciprocal signaling mechanisms in the CNS and adipose tissue to maintain organismal energy homeostasis. We are focusing on the integration of afferent signals from the periphery in neuronal populations of the mediobasal hypothalamus as well as the efferent signals from the CNS to adipose tissue and its implications for adipose tissue function. Furthermore, we are discussing central mechanisms that fine-tune the immune system in adipose tissue depots and contribute to organ homeostasis. Elucidating this complex signaling network that integrates peripheral signals to generate physiological outputs to maintain the optimal energy balance of the organism is crucial for understanding the pathophysiology of obesity and metabolic diseases such as type 2 diabetes.

## Introduction

In most mammalian species, the size of the brain and adipose depots are inversely correlated, indicating compensatory buffering strategies to protect against starvation (Navarrete et al., 2011). However, in humans, dietary and social factors have led to increased brain sizes alongside large adipose tissue stores despite substantial energetic costs (Navarrete et al., 2011; Heldstab et al., 2016). Adipose depots make up a remarkable proportion of human body mass, allowing physiological buffering and efficient partitioning of unutilized calories (Ofenheimer et al., 2020; Liu et al., 2021). The size of the human brain allows for increased cognitive flexibility, representing an additional mechanism to keep the energy intake constant and to maintain the body’s energy requirements (Heldstab et al., 2016). Therefore, a fine-tuned crosstalk between the two organs orchestrates the regulation of feeding, energy storage, and energy expenditure. Adipose depots constitute a dynamic endocrine organ secreting multiple adipokines into circulation that signal energy availability to the brain. Furthermore, sensory innervation of adipose depots allows for the detection of locally released free fatty acids and adipokines and therefore represents an additional afferent route from adipose tissue to the central nervous system (CNS) (Murphy et al., 2013; Garretson et al., 2016). The CNS integrates these peripheral signals to generate physiological responses to maintain the optimal energy balance of the organism.

This review highlights the coordinated reciprocal signaling between the CNS and white adipose tissue. We are discussing the integration of afferent signals from the periphery in neuronal populations of the mediobasal hypothalamus as well as the efferent signals from the CNS to adipose tissue and its implications for adipose tissue function. Furthermore, we are focusing on central mechanisms that regulate resident immune cell function in adipose tissue depots and subsequently contribute to organ homeostasis.

## Efferent signals from the central nervous system are crucial regulators of white adipose tissue function

The brain interacts with white adipose tissue depots through distinct efferent sympathetic nerves, releasing the catecholamine norepinephrine (NE) from their nerve terminals. In white adipose tissue (WAT), sympathetic nerve terminals are located adjacent to >90% of adipocytes, forming a dense network of sympathetic arborizations (Jiang et al., 2017). Importantly, sympathetic outflow to the adipose tissue is the principal initiator of adipose tissue lipolysis (Fredholm and Karlsson, 1970; Youngstrom and Bartness, 1995; Dodt et al., 2003; Bartness et al., 2010). Electrical stimulation of sympathetic nerve fibres in rat epididymal adipose tissue explants resulted in the rapid release of fatty acids and glycerol into the incubation medium, which provided some of the first evidence for this regulation (Correll, 1963). The necessity of WAT sympathetic nervous system (SNS) innervation for lipolysis was elegantly demonstrated by direct optogenetic activation of sympathetic inputs to adipose tissue, which was sufficient to promote a local lipolytic response and fat mass reduction (Zeng et al., 2015). It is worth noting that the extent of sympathetic innervation and outflow differs between the different depots of WAT (Youngstrom and Bartness, 1995; Brito et al., 2007; Brito et al., 2008). Furthermore, the rate of extracellular NE clearance also influences the sympathetic tone. Most NE is sequestered from the synapse through the solute carrier family six member 2 (SLC6A2) monoamine transporter expressed on sympathetic neurons. Pirzgalska et al. (2017) reported a macrophage subtype capable of dampening the sympathetic tone in adipose tissue by lowering noradrenaline bioavailability. These specialized sympathetic neuron-associated macrophages (SAMs) are able to scavenge noradrenaline through the transporter Slc6a2 and degrade it using the enzyme monoamine oxidase A (MAOa) (Camell et al., 2017). Treating aged mice with an MAOA inhibitor increased adipose tissue concentrations of norepinephrine (NE) and restored the aging-related fasting-induced lipolysis defect (Camell et al., 2017). In addition, genetic ablation of Slc6a2 was shown to be sufficient to increase NE levels in serum, which results in improved brown adipose tissue (BAT) performance and browning of WAT (Pirzgalska et al., 2017). Of note, adipocytes express the organic cation transporter 3 (Oct3; Slc22a3), allowing the clearance of NE (Ayala-Lopez et al., 2015; Song et al., 2019). This indicates that several cell types are involved in the regulation of NE bioavailability specifically in white adipose microenvironments. Collectively, the bioavailability of catecholamines, such as NE, constitutes a specific regulatory mechanism in adipose tissue homeostasis.

**FIGURE 1:**
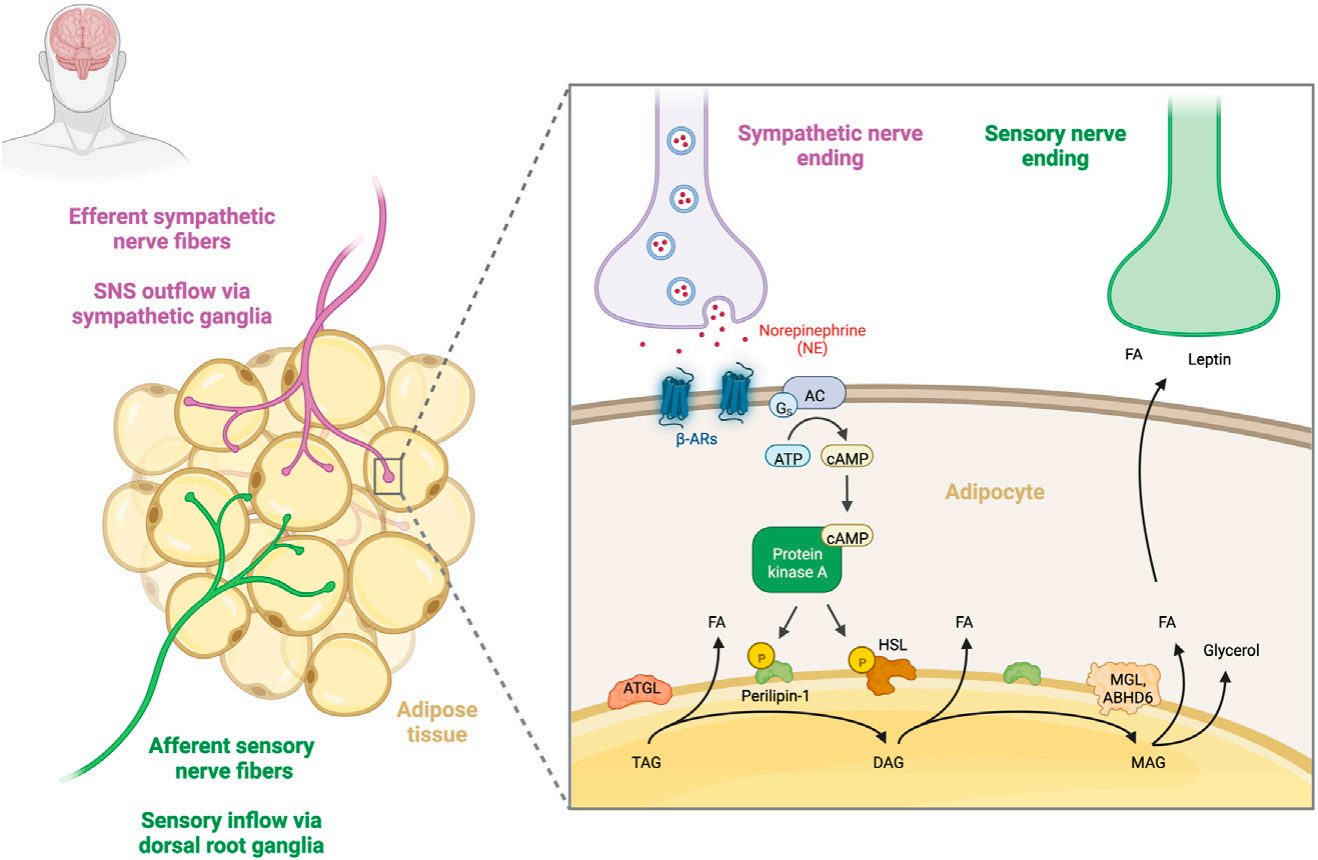
Sympathetic regulation of adipose tissue lipolysis. Sympathetic and sensory innervation of human adipose tissue. Sympathetic nerve fibres that travel from the CNS to innervate adipose tissue (purple) and sensory nerve fibres that relay information from adipose tissue to the CNS (green) are shown. Release of noradrenaline (NE) from efferent sympathetic fibres leads to the activation of β-adrenergic receptors and the subsequent dissociation of the receptor-coupled Gs protein leads to activation of adenylate cyclase (AC), which increases intracellular cAMP levels. High cAMP levels activate protein kinase A (PKA), phosphorylating hormone-sensitive lipase (HSL) and perilipin-A (PLIN1). This initiates the activation of a set of lipases, such as adipose triglyceride lipase (ATGL) and monoglyceride lipase (MGL) or α/β hydrolase-domain 6 (ABHD6), allowing for consecutive hydrolysis of TGs into fatty acids (FA) and glycerol. Increased lipolysis in turn activates WAT afferent sensory nerve endings, which are able to sense local FA and leptin concentrations (Garretson et al., 2016). Created with BioRender.com.

Stimulation of lipolysis requires the activation of G-protein–coupled *α*- and *β*-adrenoceptors (*α-* and *β-*ARs) on adipocytes (Barbe et al., 1996). The extent of the lipolytic activity depends on a balance between lipolysis stimulation by β-ARs (β1–3-AR) and lipolysis inhibition by α2-ARs [for review see (Evans et al., 2019; Collins, 2022)]. Activating β-adrenergic receptors leads to dissociation of the receptor-coupled G_s_ protein and activation of adenylate cyclase (AC), which increases intracellular cAMP levels. High cAMP levels activate protein kinase A (PKA), which phosphorylates hormone-sensitive lipase (HSL) and perilipin-A (PLIN1). This initiates a signaling cascade that leads to the activation of lipases, such as adipose triglyceride lipase (ATGL) and monoglyceride lipase (MGL) or α/β hydrolase-domain 6 (ABHD6), allowing triglycerides to be hydrolyzed sequentially into fatty acids (FA) and glycerol (Figure 1) (Grabner et al., 2021).

In order to fine-tune adipose lipolytic activity, neuropeptide Y (NPY), which is co-stored with NE, is released from sympathetic nerve terminals and inhibits lipolysis by binding to its receptor NPYR1 (Lundberg et al., 1990; Labelle et al., 1997; Bradley et al., 2005). NPY receptors are highly expressed on human adipocytes, and most abundant in subcutaneous adipose depots (Castan et al., 1993; Serradeil-Le Gal et al., 2000; Kos et al., 2007). The release of NPY on the other hand has a considerable proliferative effect on adipocyte precursors and stimulates adipogenesis (Kuo et al., 2007; Yang et al., 2008). In this context, NPY promotes the accumulation and storage of energy.

Lipolysis increases the availability of free fatty acids which in turn activate local WAT afferents that mediate acute induction of thermogenesis in distant BAT depots (Garretson et al., 2016; Nguyen et al., 2017). This data is strong indication of WAT and BAT crosstalk *via* afferent sensory feedback in order to maintain thermoregulation.

## Efferent signals from the central nervous system regulate adipose tissue expansion

In addition to its role as principal regulator of adipose tissue lipolysis, the SNS is also involved in the regulation of adipose tissue mass and plasticity. Here, the local release of NE constitutes an important negative regulator of adipogenesis (Jones et al., 1992). Surgical local denervation or chemical sympathectomy of WAT increased adipocyte precursor cell proliferation and accelerated preadipocyte differentiation (Cousin et al., 1993; Bowers et al., 2004; Foster and Bartness, 2006). However, surgical denervation of nerve bundles results in a mixed denervation of sensory and sympathetic nerve fibres that impacts the interpretation of these findings. Furthermore, transgenic mice with a deficiency of the neuronal transcription factor Nscl-2 displayed reduced nerve density in white adipose depots and this reduction in innervation was accompanied by an increase in numbers of preadipocytes (Ruschke et al., 2009). Conversely, sensory denervation (through local microinjections of capsaicin) did not affect preadipocyte proliferation and differentiation (Foster and Bartness, 2006). The inhibitory effect of NE on the proliferation of WAT adipose progenitor cell populations is likely mediated through β1-adrenergic receptors (β1-ARs). Schneider et al. (2018) demonstrated that the selective β1-AR agonist dobutamine diminished preadipocyte proliferation. Interestingly, the activation of the parasympathetic nervous system (PSNS) may play an opposing role to that of the SNS. Here, reduced melanocortin signaling due to increased vagal activity within the splanchnic compartment actively facilitates adipose tissue expansion (Holland et al., 2019). Importantly, a lack of parasympathetic innervation in the WAT has been reported and therefore further research is needed to determine how the brain-melanocortin-*vagus* efferent axis regulates fat mass gain (Giordano et al., 2006).

## Central integration of adipose tissue signals

Retrograde tracing experiments have revealed numerous brain areas that are polysynaptically connected to adipose tissue. This includes several nuclei in the mediobasal hypothalamus, such as the arcuate nucleus (ARC), the ventromedial hypothalamus (VMH), the dorsomedial hypothalamus (DMH), the lateral hypothalamus (LH) and the paraventricular nucleus of the hypothalamus (PVH) (Bamshad et al., 1998; Ryu and Bartness, 2014). These nuclei contain several functionally distinct neuronal populations that adapt integrative physiology to the organismal energy state (Aponte et al., 2011; Brandt et al., 2018; Chen et al., 2015; Sutton and Krashes, 2020; van den Top et al., 2004; Zhan et al., 2013). These neurons express high levels of receptors that allow for the integration of peripheral metabolic feedback signals within the CNS in order to generate physiological output (Jais and Bruning, 2022). This output is conveyed as sympathetic outflow to adipose tissue (and other peripheral organs) (Guilherme et al., 2019).

The most well-defined neurocircuit in the context of integrative physiology is the melanocortin system, which consists of the functionally antagonistic anorexigenic proopiomelanocortin (POMC)-expressing neurons and the orexigenic agouti-related peptide (AgRP)-expressing neurons in the arcuate nucleus (ARC) of the mediobasal hypothalamus (Gautron et al., 2015). POMC neurons are activated by energy surplus and inhibit food intake after prolonged periods of feeding (Aponte et al., 2011). These neurons release the melanocortin α-melanocyte-stimulating hormone (α-MSH) into the paraventricular nucleus (PVH) where it binds to the melanocortin receptor 4 (MC4R), resulting in reduced food intake and increased energy expenditure (Cone, 2006; Zhan et al., 2013; Krashes et al., 2016). AgRP neurons are located adjacent to the third ventricle in close proximity to the median eminence (ME), allowing them to sense peripheral metabolic signals. A negative energy balance increases AgRP neuronal excitability, which is rapidly suppressed upon the initiation of feeding (Hahn et al., 1998; Aponte et al., 2011; Betley et al., 2015). Mechanistically, AgRP acts as an inverse agonist for the melanocortin receptor 4 (MC4R) that competes with α-MSH released from POMC neurons for binding sites on the MC4R (Haskell-Luevano and Monck, 2001). AgRP neurons also release neuropeptide Y (NPY) as well as the inhibitory neurotransmitter gamma-aminobutyric acid (GABA), and the promotion of feeding depends on both NPY and GABA release from these cells (Tong et al., 2008; Krashes et al., 2013).

Importantly, both AgRP and POMC neurons express receptors for leptin, insulin and other energy-state communicating hormones and are therefore subject to feedback regulation (Belgardt and Bruning, 2010; Varela and Horvath, 2012; Vogt and Bruning, 2013; Biglari et al., 2021; Deem et al., 2022).

### Leptin signaling in the mediobasal hypothalamus

The discovery of leptin revealed a central mechanism of how adipose tissue communicates with the CNS (Zhang et al., 1994; Halaas et al., 1995; Montague et al., 1997). Leptin is a hormone primarily released by WAT proportional to the size of fat stores and central leptin signaling is an essential regulator of lipid storage (Frederich et al., 1995; Klein et al., 1996; Fruhbeck et al., 1998). The lipolytic effects of leptin are mediated by neuronal pathways as evidenced by the fact that selective denervation within WAT depots prevents the lipolysis-increasing effects of leptin (Buettner et al., 2008; Zeng et al., 2015). Several studies have identified POMC neurons in the ARC as potential regulators of lipolysis (Kaushik et al., 2012; Shin et al., 2017; Gomez-Valades et al., 2021). For example, the loss of autophagy in POMC neurons decreases α-melanocyte-stimulating hormone (MSH) levels, which in turn leads to impaired lipolysis (Kaushik et al., 2012). Leptin directly activates POMC neurons through the leptin receptor (LEPR) (Cowley et al., 2001; Balthasar et al., 2004). In addition, leptin reduces the inhibitory tone of presynaptic GABAergic neurons to postsynaptic POMC neurons (Vong et al., 2011). Mechanistically, leptin signaling in the mediobasal hypothalamus increases adipose tissue lipolysis by post-translational regulation of hormone-sensitive lipase (HSL) in adipocytes (Buettner et al., 2008). In this regard, several nuclei of the mediobasal hypothalamus, such as the ARC, VMH, DMH, LH, and PVH are mediating leptin action on SNS activity (Rahmouni and Morgan, 2007; Simonds et al., 2012; Harlan and Rahmouni, 2013; Shi et al., 2020). Moreover, leptin-stimulated central PI3K signaling regulates energy expenditure through activation of SNS activity to WAT leading to browning of adipocytes and increased energy expenditure (Plum et al., 2007). Sympathetic nerve fibers in white adipose tissue establish neuro-adipose junctions, thereby allowing for the local regulation of lipolysis (Zeng et al., 2015). Leptin also regulates the plasticity of the sympathetic innervation of adipose tissue. Here, a population of BDNF-expressing neurons in the paraventricular nucleus of the hypothalamus (BDNF^PVH^) dynamically regulates the sympathetic innervation downstream of leptin-sensitive AGRP and POMC neurons in the ARC (Wang et al., 2020).

*De novo* lipogenesis, the process of converting carbohydrates into fatty acids, is regulated by hypothalamic leptin signaling as demonstrated by acute infusion of leptin into the mediobasal hypothalamus, which potently suppresses key *de novo* lipogenic enzymes (Buettner et al., 2008). Interestingly, *de novo* lipogenesis in adipocytes might provide regulatory feedback for sympathetic neuronal activity as the suppression of the key lipogenic enzyme fatty acid synthase (FASN) in white adipocytes enhances sympathetic activity (Guilherme et al., 2017). Furthermore, leptin regulates its own expression in adipocytes through a SNS-dependent mechanism that requires NE. Acute treatment of mice with β3-adrenoceptor agonists suppresses leptin secretion from adipocytes through a β3-adrenergic receptor (β3-AR)–cAMP-dependent mechanism (Trayhurn et al., 1996). Conversely, systemic inhibition of catecholamine synthesis in rats increased plasma leptin levels by 15-fold (Sivitz et al., 1999).

### Insulin signaling in the mediobasal hypothalamus

Another important signaling molecule in the CNS-adipose crosstalk involving melanocortin neurons is insulin. Insulin is an anabolic peptide hormone secreted by β-cells of the pancreas and insulin signaling in the mediobasal hypothalamus dampens sympathetic nerve activity to adipose tissue, suppresses lipolysis and allows for increased adipose tissue retention of fatty acids (Scherer et al., 2011; Shin et al., 2017). Furthermore, this reduction in lipolysis reduces hepatic glucose production by limiting the flux of energy substrates necessary for gluconeogenesis (Scherer et al., 2011). Importantly, genetic disruption of the insulin receptor (IR) on POMC neurons resulted in impaired suppression of adipose tissue lipolysis (Shin et al., 2017). However, POMC neurons are a heterogeneous cell population (Dodd et al., 2018; Biglari et al., 2021). The phosphatase TCPTP mediates insulins effects on POMC neurons as elevated expression of the phosphatase TCPTP in POMC neurons represses insulin signaling. Conversely, a decreased expression of TCPTP enhances insulin signaling and therefore the proportion of POMC neurons activated by insulin (Dodd et al., 2018). However, the effects of POMC-specific TCPTP expression on the regulation of lipolysis are still unknown.

### Sensing of free fatty acids in the mediobasal hypothalamus

Various cell types in the mediobasal hypothalamus are capable of sensing circulating fatty acids. Tanycytes, specialized ependymal glia cells that line the wall of the third ventricle near the ARC, sense free fatty acids and subsequently regulate WAT lipolysis through hypothalamic FGF21 signaling (Geller et al., 2019). Importantly, long-chain fatty acids (LCFA) such as oleic acid (OA) are able to excite a subset of POMC neurons directly through inhibition of ATP-activated potassium (KATP) channels (Jo et al., 2009). Moreover, central administration of a MC4R antagonist abolished the (anorexigenic) actions of OA (Schwinkendorf et al., 2011).

These findings indicate that the melanocortin system acts as a signaling hub for regulating WAT lipolysis. Indeed, SNS outflow neurons to WAT express melanocortin-4 receptor (MC4R) mRNA (Song et al., 2005). Pharmacological activation of MC3/4R in the CNS stimulates lipolysis independent of food intake (Nogueiras et al., 2007). Furthermore, central infusion with MC3/4R agonists provokes differential sympathetic drives to various adipose tissue depots (Brito et al., 2007).

It is worth noting that peripheral signals are relayed not only *via* the circulation, but also through sensory innervation of adipose depots. Sensory nerve endings allow for the detection of local leptin levels directly in the adipose tissue depot (Fishman and Dark, 1987; Niijima, 1998, 1999). Therefore, the integration of information from leptin reaching the brain *via* the circulation and information about individual adipose tissue depots leptin levels *via* sensory fibers might help to adjust the sympathetic tone from the CNS in an adipose tissue depot-specific manner.

In conclusion, these data clearly highlight the importance of the melanocortin system in the CNS-adipose crosstalk. Further efforts are needed however to understand the deregulation of these signaling networks during the development of leptin and insulin resistance.

## Resident immune cells contribute to adipose tissue homeostasis through multiple mechanisms

Immune mechanisms in the adipose tissue have been widely studied within the onset of obesity and insulin resistance (Pekala et al., 1983; Wellen and Hotamisligil, 2003; Lumeng et al., 2007a; Lumeng et al., 2007b). Nevertheless, adipose tissue contains resident immune cells that maintain organ homeostasis. Adipose tissue macrophages (ATMs) are the most abundant immune cell type, which occupy up to 10% of stromal cells under a steady-state and are usually uniformly distributed (Weisberg et al., 2003). ATMs help maintain adipose tissue homeostasis by controlling key signaling pathways involved in adipogenesis, lipogenesis, lipolysis, and lipid uptake (Kosteli et al., 2010; Bilkovski et al., 2011; Nguyen et al., 2011; Brunner et al., 2020; Chen et al., 2021). In addition, ATMs play a critical role in the vascular homeostasis of the organ as an adequate blood flow is essential for adipose tissue expansion and metabolic functions. This was demonstrated by co-culturing macrophages and adipocytes, which lead to increased expression of VEGFA and other pro-angiogenic factors (Yadav et al., 2020). Interestingly, VEGFA exerts potent neurotrophic and synaptotrophic effects as well (Sondell et al., 1999; Pelletier et al., 2015; Calvo et al., 2018). However, the role of VEGFA in regulating adipose tissue innervation has not been explored to date. In addition, the comparison of adipose tissue obtained from wildtype and ATM-depleted mice revealed that ATMs play a pivotal role in suppressing the expression of pro-inflammatory cytokines (Chen et al., 2021).

### Bi-directional crosstalk between adipose tissue-resident immune cells and nerve fibres

Extensive crosstalk between the SNS and resident ATMs regulates adipose tissue homeostasis and the activation of β-adrenergic signaling is a powerful regulator of adipose tissue function. Adrenergic receptors are expressed in macrophages, particularly β2-adrenergic receptors (β2-AR) (Pavlov et al., 2018; Petkevicius et al., 2021). ATMs from mice treated with CL316,243 (a β-adrenergic agonist) increased beige adipogenesis significantly (Lee et al., 2016). This process entails triggering the death of unilocular white adipocytes, clearance of dead cells, and the recruitment of UCP1+ adipocyte progenitors and further differentiation (Lee et al., 2016). Moreover, β2-AR stimulation was also shown to be essential for maintaining low TNFα expression levels in ATMs in lean mice and for M2 macrophage polarization (Grailer et al., 2014; Tang et al., 2015). Interestingly, Petkevicius et al. (2021) have recently shown that macrophage β2-AR activation is dispensable for the development of metabolic inflammation. However, nerve-associated macrophages can be found in several tissues throughout the body, where they are involved in regulation of metabolic homeostasis potentially utilizing different signaling mechanisms [for review see (Kolter et al., 2020)]. Therefore, further studies on the function of nerve-associated macrophages in metabolic homeostasis are required.

**FIGURE 2:**
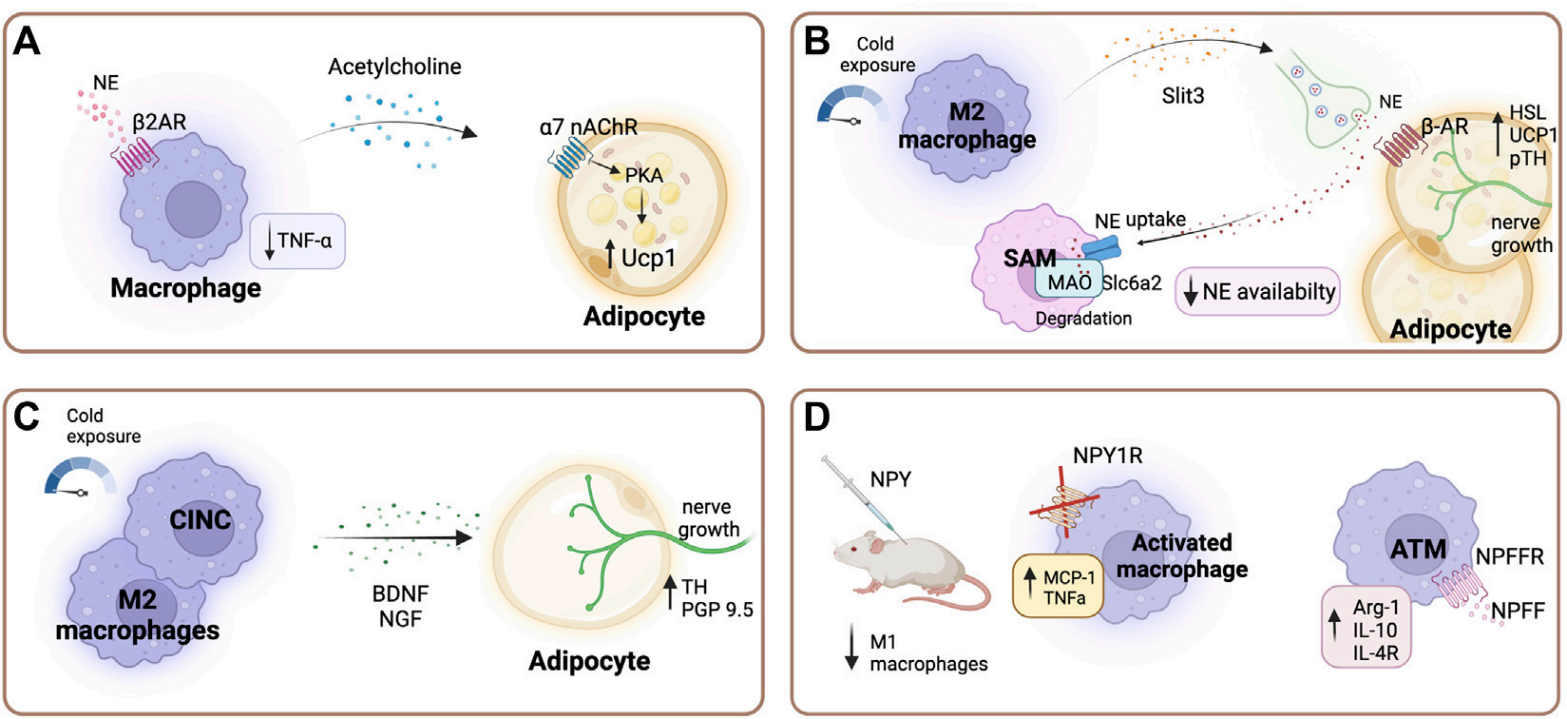
Sympathetic regulation of adipose tissue macrophages. **(A)** Beta-2 adrenergic receptor (β2-AR) stimulation in macrophages promotes the production and secretion of acetylcholine, which acts on adipocytes *via* acetylcholine receptors, stimulating thermogenesis through the PKA pathway and consequently inducing thermogenic gene expression (Knights et al., 2021; Meng et al., 2021). Additionally, β2-AR stimulation is essential for maintaining low tumor necrosis factor -alpha (TNFα) levels (Wang et al., 2003). **(B)** After cold stimulation M2 macrophages secrete cytokine Slit3, which promotes sympathetic nerve growth pathway, and stimulates the synthesis and release of norepinephrine (NE), subsequently inducing thermogenesis (Wang et al., 2021). Specialized sympathetic neuron-associated macrophages (SAMs) scavenge noradrenaline through the transporter Slc6a2 and degrade it using the enzyme monoamine oxidase A (MAOa). Thereby regulating local adipose tissue availability of NE (Camell et al., 2017; Pirzgalska et al., 2017). **(C)** A subset of macrophages belonging to the cold-induced neuroimmune cells (CINCs) and M2 macrophages secrete neurotrophic factors such as brain-derived neurotrophic factor (BDNF) upon cold exposure, promoting adipocyte nerve growth (Blaszkiewicz et al., 2022; Xie et al., 2022). **(D)** Neuropeptide Y (NPY) modulates inflammatory response in macrophages. NPY supplementation in lean mice leads to a decreased number of M1 adipose tissue macrophages (ATMs) (Singer et al., 2013). Deficiency of NPY1 receptor increases the secretion of TNFα and monocyte chemoattractant protein-1 (MCP-1) under inflammatory conditions (Macia et al., 2012). Neuropeptide FF receptor 2 (NPFFR2) is predominantly expressed in ATMs compared to other macrophage populations. In ATMs neuropeptide FF (NPFF) increases arginase 1, interleukin (Il-) 10, and Il-4 receptor expression (Waqas et al., 2017). Created with BioRender.com.

Furthermore, β2-AR stimulation in macrophages promotes thermogenesis *via* the production and secretion of acetylcholine, which acts on adipocytes *via* acetylcholine receptors, stimulating the PKA pathway and subsequently inducing thermogenic gene expression (Figure 2A) (Knights et al., 2021; Meng et al., 2021).

In addition, cholinergic signaling in macrophages through the alpha-7 nicotinic acetylcholine receptor plays an anti-inflammatory role by inhibiting TNFα release (Wang et al., 2003). TNFα has long been implicated in development of insulin resistance (Hotamisligil et al., 1993), and reduced TNFα activity improves systemic insulin resistance (Togashi et al., 2002). Exposure to low-dose TNFα impairs central insulin and leptin signaling (Romanatto et al., 2007). Furthermore, TNFα induces the expression of PTP1B in the ARC through NF-κB activation (Zabolotny et al., 2008). This represses insulin signaling and therefore lowers the proportion of POMC neurons activated by insulin.

Furthermore, through the secretion of the cytokine Slit3 upon cold exposure ATMs exert a modulatory function in the sympathetic innervation of adipose tissue. Slit3 promotes sympathetic nerve growth *via* the Slit-ROBO pathway, and additionally stimulates the synthesis and release of NE, which in turn promotes thermogenesis (Figure 2B) (Wang et al., 2021). A subset of macrophages, cold-induced neuroimmune cells (CINCs), are recruited to the tissue in response to cold stimulation and have shown to coordinate gene expression involved in nerve survival and plasticity (Blaszkiewicz et al., 2022). These cells secrete neurotrophic factors such as brain-derived neurotrophic factor (BDNF), which has been shown to exert an important role in WAT innervation (Blaszkiewicz et al., 2020; Wang et al., 2020) (Figure 2C). For instance, the knockout of BNDF in myeloid lineage cells in mice leads to decreased innervation of inguinal subcutaneous WAT depots (Blaszkiewicz et al., 2020). In addition, the TrkB receptor (a receptor with high binding affinity for BDNF) was shown to be expressed on sensory and sympathetic nerve fibers in subcutaneous WAT (Blaszkiewicz et al., 2022). Moreover, hypothalamic BDNF overexpression in DIO and lean mice lead to an up-regulation of β-ARs and UCP-1 in the WAT (Cao et al., 2009; Cao et al., 2011). Whereas heterozygous BDNF knockout mice (mice with approximately 40% less BDNF protein than wild type control animals) showed a selective suppression of β-ARs in WAT (Cao et al., 2011).

### Neuropeptide Y regulates the function of ATMs

NPY potently influences metabolic function in peripheral tissue and has been shown to play a role in the inflammatory response modulation of ATMs (Ruohonen et al., 2008; Singer et al., 2013). ATMs express Y1, Y2, and Y5 receptors and the *in vitro* blockade of these receptors enhances the expression of pro-inflammatory genes (Singer et al., 2013). The Y1 receptor expression in immune cells of adipose tissue depots was critical in controlling inflammation and insulin resistance in obesity (Figure 2D) (Macia et al., 2012). In this study, the authors investigated periovarian adipose depots from female mice. Moreover, *in vivo* NPY supplementation decreased M1 proinflammatory macrophages in lean mice (Singer et al., 2013). Park et al. (2021) demonstrated that NPY secreted by macrophages also upregulates adipogenic and lipogenic gene expression profiles. Nevertheless, obese mice show higher levels of circulating NPY, and increased expression of NPY and NPY 2-receptor (NPY2R) mRNA in subcutaneous adipose tissue, indicating that circulating NPY originates from adipose tissue (Kuo et al., 2007). Additionally, Kuo et al. reported that stress in mice leads to the release of NPY from sympathetic nerve fibres in WAT and activation of NPY2R, which stimulates macrophage infiltration and a metabolic syndrome-like condition (Kuo et al., 2007). NPY function is believed to be determined by site-specific NPY and NPY receptor expression. In lean animals, NPY is expressed in non-ATMs, such as adipocytes, as well as ATMs. However, during obesity NPY expression is significantly induced in ATMs (Schwarz et al., 1994; Kos et al., 2007; Singer et al., 2013). However, the contribution of NPY release from sympathetic nerve endings to the total adipose tissue NPY levels has not been investigated to date. Hence, further studies are needed to clarify the exact role of adipose tissue-specific NPY in obesity and insulin resistance development.

### Neuropeptide FF effects on adipose tissue

Furthermore, neuropeptide FF (NPFF) has been shown to decrease food intake in mice and inhibit adipocyte development (Murase et al., 1996; Herrera-Herrera and Salazar-Olivo, 2008; Ruohonen et al., 2008). NPFF is potentially released from nerve endings in adipose tissue, although this has not been demonstrated so far (van Harmelen et al., 2010). This neuropeptide shows potent effects in the regulation of ATM function. In isolated ATMs, NPFF treatment increased arginase 1, IL-10, and IL-4R expression and in mice NPFF treatment improved glucose tolerance and insulin sensitivity (Figure 2D) (Waqas et al., 2017). Neuropeptide FF receptor 2 (NPFFR2) is predominantly expressed in ATMs compared to other macrophage populations (Waqas et al., 2017). Moreover, sustained exposure to NPFF can increase ATMs numbers more effectively than IL-4, a cytokine known to induce M2 activation (Waqas et al., 2017). Interestingly, hypothalamic NPFF signaling through the NPFFR2 receptor plays a key role in mediating diet-induced adaptative thermogenesis as evidenced by an impaired BAT response in Npffr2 knockout mice (Zhang et al., 2018).

Melanocortins also play an important anti-inflammatory role through the activation of melanocortin receptors (MCRs) expressed in adipose tissue resident immune cells (Wang et al., 2019). Several *in vivo* and *in vitro* studies have shown anti-inflammatory effects mediated by melanocortin agonists acting on macrophages (Wang et al., 2019). For example, Getting et al. (1999) reported that MC3/4R activation in peritoneal macrophages reduces the release of pro-inflammatory cytokines. In addition, α-melanocyte-stimulating hormone (α-MSH) was shown to inhibit the production of nitric oxide and NF-κB nuclear translocation in cultured macrophages (Star et al., 1995; Rajora et al., 1996; Mandrika et al., 2001). Activation of POMC neurons in the ARC and the anterior pituitary leads to the release of α-MSH into the circulation and subsequent activation of MCRs in the adipose tissue (Elias et al., 2000; Cowley et al., 2001). However, the POMC gene is expressed in various immune cells (residing within the adipose tissue compartment) as well (Lyons and Blalock, 1997; Blalock, 1999). These local effects of adipose tissue-derived peptide products of the POMC gene need further investigation. Of note, in both mouse and human preadipocytes α-MSH inhibits proliferation and in adipocytes it decreases the expression and secretion of leptin (Smith et al., 2003; Hoggard et al., 2004).

It is clear that ATMs impact the innervation of adipose tissue in numerous ways and vice versa the adipose tissue innervation directly impacts ATM functions *via* neurotransmitter release.

**FIGURE 3:**
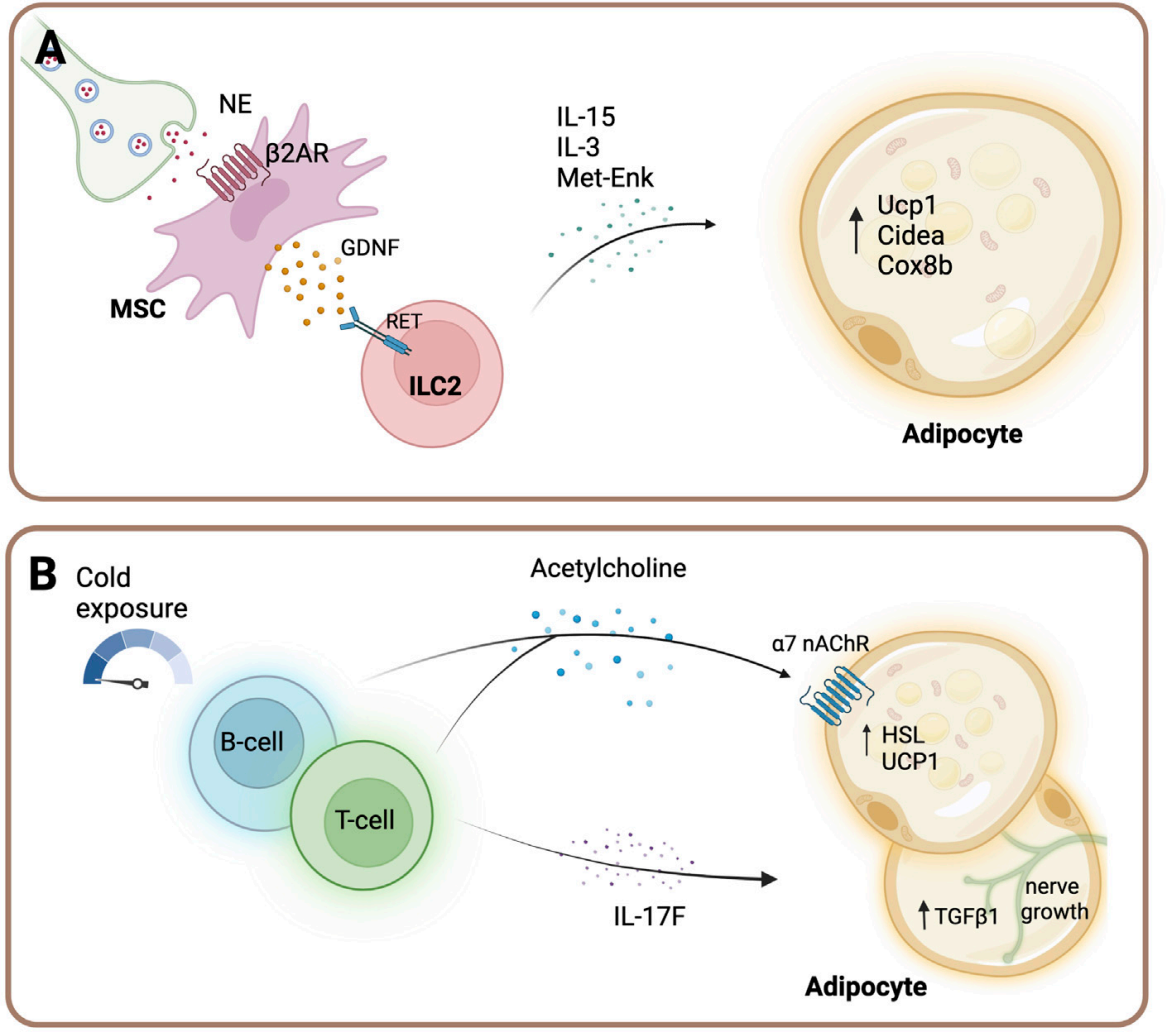
Sympathetic regulation of immune cells in adipose tissue. **(A)** Sympathetic outflow acts on β2-AR of mesenchymal cells (MSC), which release glial-derived neurotrophic factor (GDNF). GDNF in turn activates group 2 innate lymphoid cells (ILC2) cells *via* the receptor RET. Activated ILC2 cells secrete interleukin (IL-)5, IL-13 cytokines and Met-enkephalin (Met-enk) and subsequently regulate adipocyte function and energy expenditure (Cardoso et al., 2021). **(B)** B cells and T cells release acetylcholine upon acute cold exposure in inguinal white adipose tissue (Jun et al., 2018). Furthermore, Gamma delta (yδ) T cells maintain sympathetic innervation of adipose tissue by driving the expression of transforming growth factor beta-1 (TGFβ1) in adipocytes *via* the IL-17F effector cytokine (Hu et al., 2020). Created with BioRender.com.

Furthermore, several other adipose tissue immune cell types are subject to sympathetic regulation. For instance, group 2 innate lymphoid cells (ILC2) were recently shown to be indirectly regulated by the SNS (Figure 3A). Activation of β2-ARs on mesenchymal stromal cells (MSC) leads to the secretion of glial-derived neurotrophic factor (GDNF) from these cells (Cardoso et al., 2021). Subsequently, GDNF acts on ILC2 cells *via* the neuroregulatory receptor RET, which ultimately leads to an increased cytokine secretion [such as interleukin (IL-)5, IL-13 and Met-enkephalin], thereby regulating adipocyte function and energy expenditure (Cardoso et al., 2021). Mice with RET receptor gain-of-function display improved glucose tolerance, decreased adipocyte size and increased UCP1 expression (Cardoso et al., 2021). Notably, sympathetic tone is required for ILC2 cells, since sympathetic denervation results in significantly suppressed ILC2 function (Ding et al., 2016).

The neurotransmitter acetylcholine (ACh) is an important contributor to immune cell function [for review see (Fujii et al., 2017) and (Cox et al., 2020)] and B cells and T cells are important (non-neuronal) acetylcholine-producing cells upon acute cold exposure in the inguinal WAT (Figure 3B) (Jun et al., 2018). Mice with hematopoietic ablation of the gene encoding for choline acetyltransferase (the rate-limiting enzyme that mediates the biosynthesis of acetylcholine) show thermogenic defects. Furthermore, the expression of the beige-fat-specific cholinergic receptor alpha two subunit (Chrna2) correlated with the local acetylcholine production and its activation was shown to be mediated in a paracrine manner (Jun et al., 2018). Furthermore, T cells, specifically yδ T cells, play a key role in the maintenance of sympathetic innervation of adipose tissue by driving the expression of TGFβ1 in adipocytes *via* the IL-17F effector cytokine (Hu et al., 2020). TGFβ1 possesses neurotrophic activity and promotes sympathetic innervation (Hu et al., 2020). Collectively, these data indicate that immune cells in adipose tissue closely associate with the SNS to maintain tissue homeostasis.

## Concluding remarks

Understanding the complex signaling networks that integrate energy availability signals from adipose tissue in the CNS to generate physiological outputs is crucial for understanding the pathophysiology of obesity and metabolic diseases such as type 2 diabetes. The findings discussed in this review clearly highlight the importance of the melanocortin system in the CNS-adipose crosstalk. However, specific neuronal populations in the mediobasal hypothalamus modify the activity of melanocortin neurons. Defining the exact molecular nature of these regulatory neurons has proven challenging. Owing to their structural and functional diversity, our current understanding of the neurocircuits involved in the control of adipose tissue is still limited. Recent technical advances in neuroscience have led to the possibility of identifying and characterizing the neurocircuits involved in the control of adipose tissue homeostasis. Identifying druggable targets on these specific neuronal populations is a prerequisite for developing novel interventions and therapeutic approaches for obesity and associated metabolic diseases.
